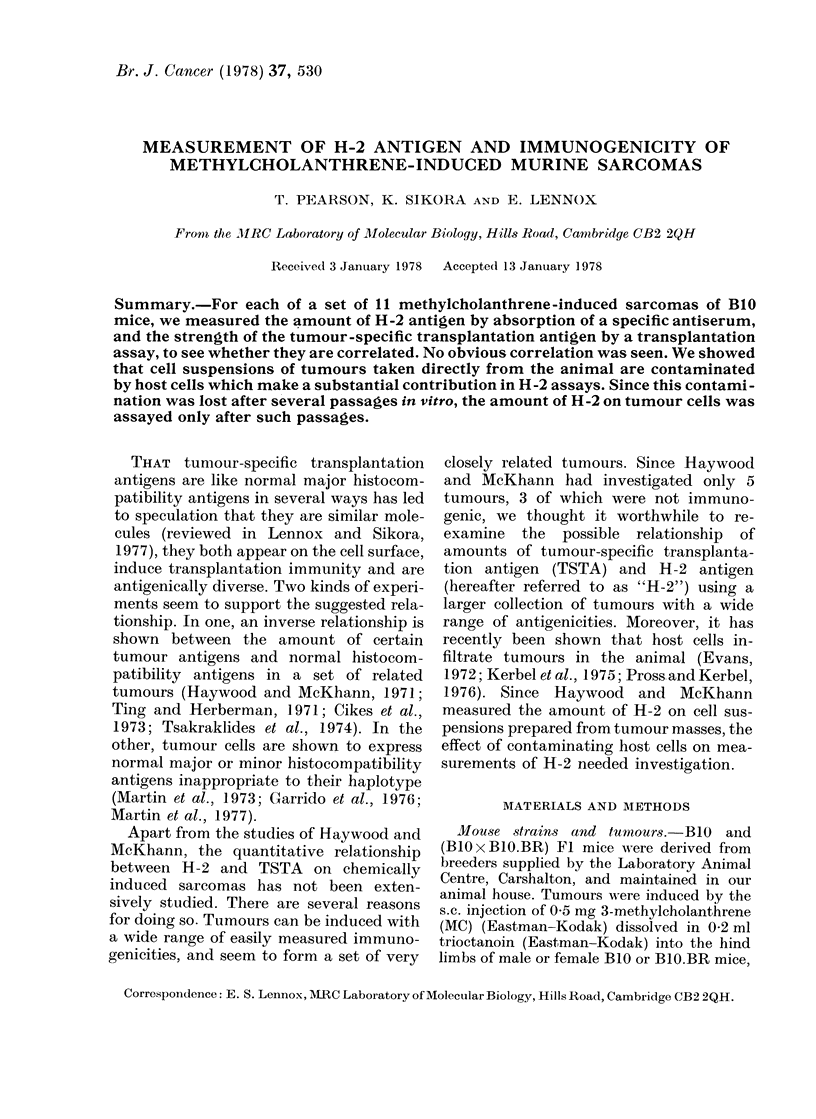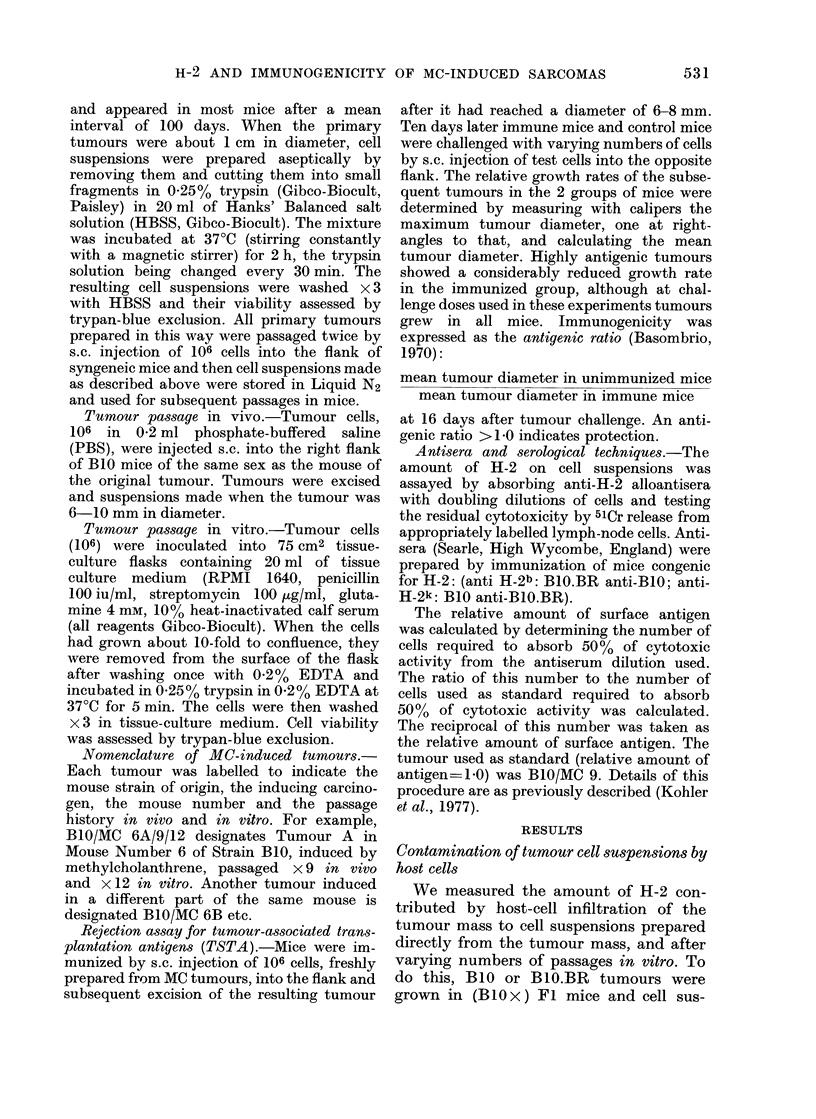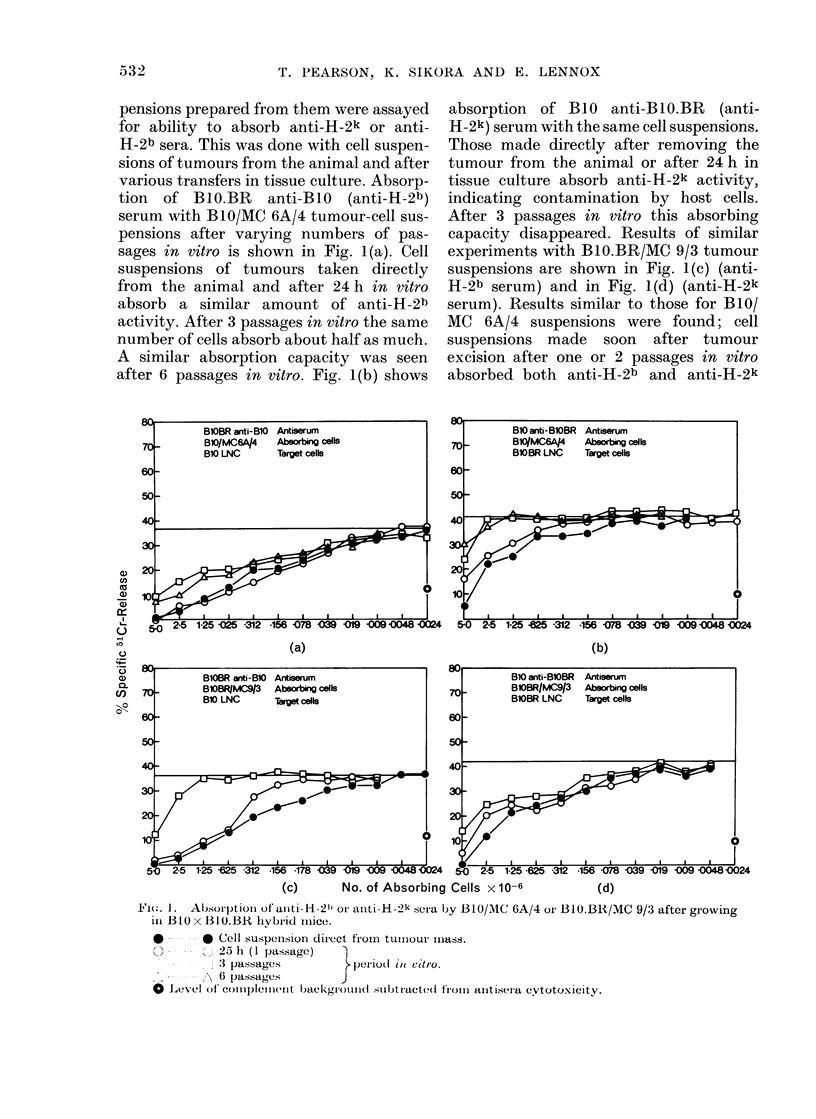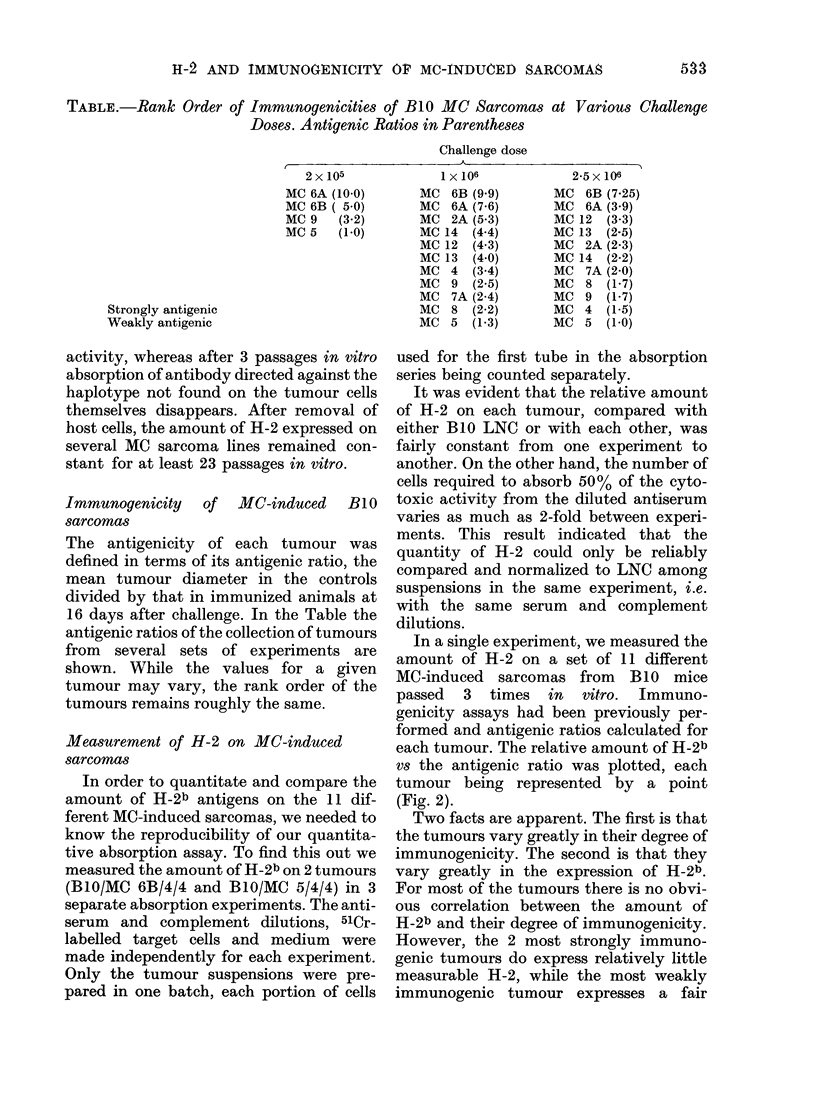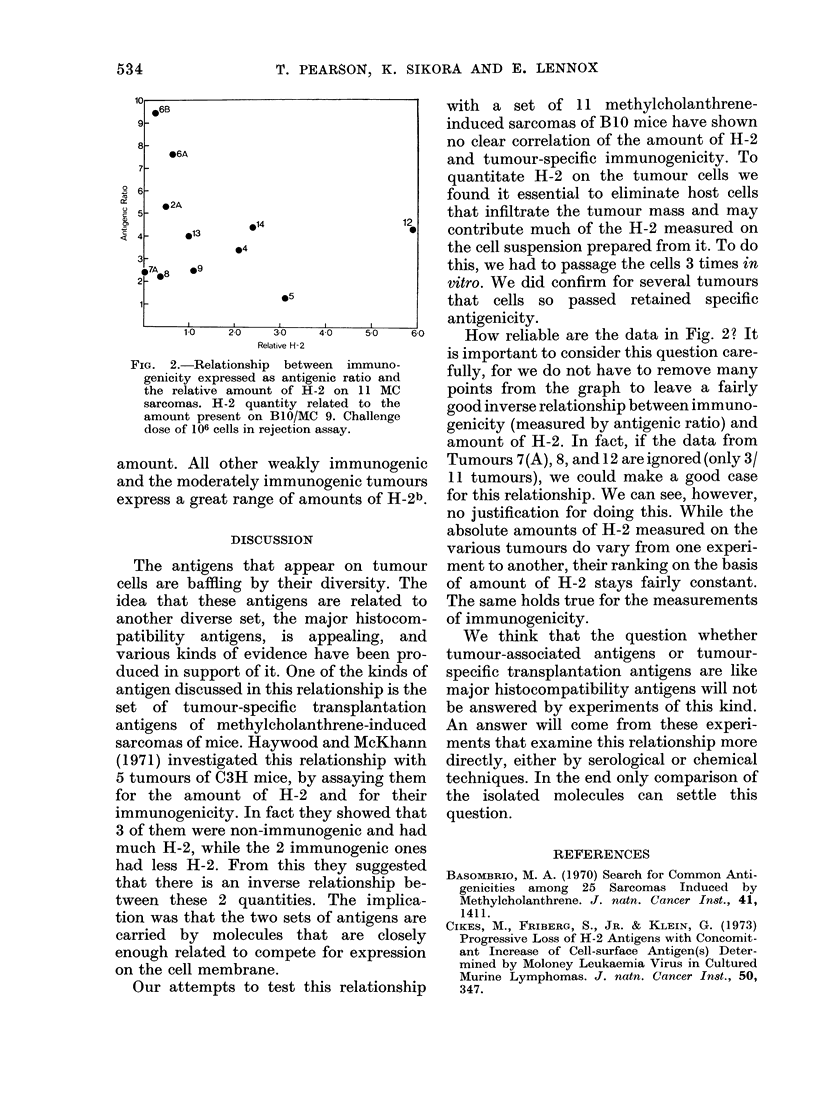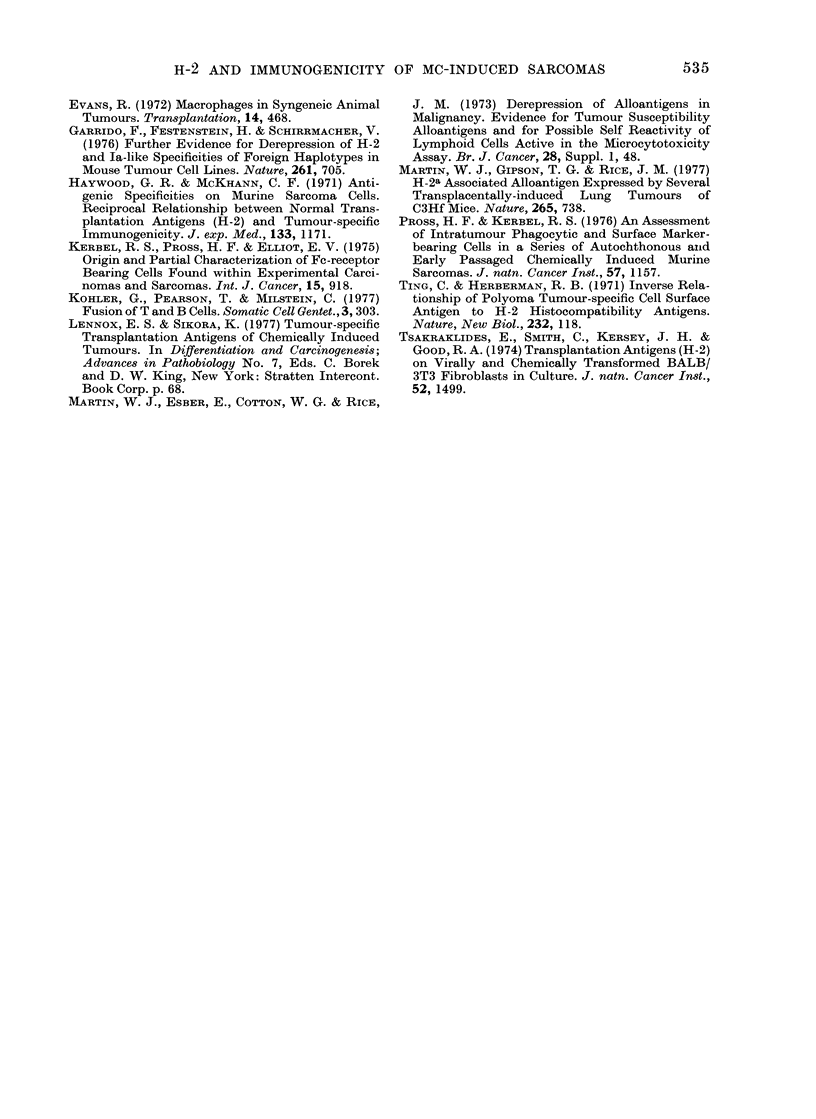# Measurement of H-2 antigen and immunogenicity of methylcholanthrene-induced murine sarcomas.

**DOI:** 10.1038/bjc.1978.81

**Published:** 1978-04

**Authors:** T. Pearson, K. Sikora, E. Lennox

## Abstract

For each of a set of 11 methylcholanthrene-induced sarcomas of B10 mice, we measured the amount of H-2 antigen by absorption of a specific antiserum, and the strength of the tumour-specific transplantation antigen by a transplantation assay, to see whether they are correlated. No obvious correlation was seen. We showed that cell suspensions of tumours taken directly from the animal are contaminated by host cells which make a substantial contribution in H-2 assays. Since this contamination was lost after several passages in vitro, the amount of H-2 on tumour cells was assayed only after such passages.


*Br. J. Cancer* (1978) **37**, 530

### MEASUREMENT OF H-2 ANTIGEN AND IMMUNOGENICITY OF METHYLCHOLANTHRENE-INDUCED MURINE SARCOMAS

T. PEARSON, K. SIKORA AND E. LENNOX

*From the MRC Laboratory of Molecular Biology, Hills Road, Cambridge CB2 2QH*

Received 3 January 1978 Accepted 13 January 1978

**Summary.—For each of a set of 11 methylcholanthrene-induced sarcomas of B10 mice, we measured the amount of H-2 antigen by absorption of a specific antiserum, and the strength of the tumour-specific transplantation antigen by a transplantation assay, to see whether they are correlated. No obvious correlation was seen. We showed that cell suspensions of tumours taken directly from the animal are contaminated by host cells which make a substantial contribution in H-2 assays. Since this contamination was lost after several passages *in vitro*, the amount of H-2 on tumour cells was assayed only after such passages.**

THAT tumour-specific transplantation antigens are like normal major histocompatibility antigens in several ways has led to speculation that they are similar molecules (reviewed in Lennox and Sikora, 1977), they both appear on the cell surface, induce transplantation immunity and are antigenically diverse. Two kinds of experiments seem to support the suggested relationship. In one, an inverse relationship is shown between the amount of certain tumour antigens and normal histocompatibility antigens in a set of related tumours (Haywood and McKhann, 1971; Ting and Herberman, 1971; Cikes *et al.*, 1973; Tsakraklides *et al.*, 1974). In the other, tumour cells are shown to express normal major or minor histocompatibility antigens inappropriate to their haplotype (Martin *et al.*, 1973; Garrido *et al.*, 1976; Martin *et al.*, 1977).

Apart from the studies of Haywood and McKhann, the quantitative relationship between H-2 and TSTA on chemically induced sarcomas has not been extensively studied. There are several reasons for doing so. Tumours can be induced with a wide range of easily measured immunogenicities, and seem to form a set of very closely related tumours. Since Haywood and McKhann had investigated only 5 tumours, 3 of which were not immunogenic, we thought it worthwhile to re-examine the possible relationship of amounts of tumour-specific transplantation antigen (TSTA) and H-2 antigen (hereafter referred to as "H-2") using a larger collection of tumours with a wide range of antigenicities. Moreover, it has recently been shown that host cells infiltrate tumours in the animal (Evans, 1972; Kerbel *et al.*, 1975; Pross and Kerbel, 1976). Since Haywood and McKhann measured the amount of H-2 on cell suspensions prepared from tumour masses, the effect of contaminating host cells on measurements of H-2 needed investigation.

### MATERIALS AND METHODS

*Mouse strains and tumours.*—B10 and (B10×B10.BR) F1 mice were derived from breeders supplied by the Laboratory Animal Centre, Carshalton, and maintained in our animal house. Tumours were induced by the s.c. injection of 0·5 mg 3-methylcholanthrene (MC) (Eastman–Kodak) dissolved in 0·2 ml trioctanoin (Eastman–Kodak) into the hind limbs of male or female B10 or B10.BR mice,

Correspondence: E. S. Lennox, MRC Laboratory of Molecular Biology, Hills Road, Cambridge CB2 2QH.

and appeared in most mice after a mean interval of 100 days. When the primary tumours were about 1 cm in diameter, cell suspensions were prepared aseptically by removing them and cutting them into small fragments in 0·25% trypsin (Gibco-Biocult, Paisley) in 20 ml of Hanks' Balanced salt solution (HBSS, Gibco-Biocult). The mixture was incubated at 37°C (stirring constantly with a magnetic stirrer) for 2 h, the trypsin solution being changed every 30 min. The resulting cell suspensions were washed ×3 with HBSS and their viability assessed by trypan-blue exclusion. All primary tumours prepared in this way were passaged twice by s.c. injection of $10^6$ cells into the flank of syngeneic mice and then cell suspensions made as described above were stored in Liquid $N_2$ and used for subsequent passages in mice.

*Tumour passage* in vivo.—Tumour cells, $10^6$ in 0·2 ml phosphate-buffered saline (PBS), were injected s.c. into the right flank of B10 mice of the same sex as the mouse of the original tumour. Tumours were excised and suspensions made when the tumour was 6—10 mm in diameter.

*Tumour passage* in vitro.—Tumour cells ($10^6$) were inoculated into 75 $cm^2$ tissue-culture flasks containing 20 ml of tissue culture medium (RPMI 1640, penicillin 100 iu/ml, streptomycin 100 μg/ml, glutamine 4 mM, 10% heat-inactivated calf serum (all reagents Gibco-Biocult). When the cells had grown about 10-fold to confluence, they were removed from the surface of the flask after washing once with 0·2% EDTA and incubated in 0·25% trypsin in 0·2% EDTA at 37°C for 5 min. The cells were then washed ×3 in tissue-culture medium. Cell viability was assessed by trypan-blue exclusion.

*Nomenclature of MC-induced tumours.*—Each tumour was labelled to indicate the mouse strain of origin, the inducing carcinogen, the mouse number and the passage history *in vivo* and *in vitro*. For example, B10/MC 6A/9/12 designates Tumour A in Mouse Number 6 of Strain B10, induced by methylcholanthrene, passaged ×9 *in vivo* and ×12 *in vitro*. Another tumour induced in a different part of the same mouse is designated B10/MC 6B etc.

*Rejection assay for tumour-associated transplantation antigens* (*TSTA*).—Mice were immunized by s.c. injection of $10^6$ cells, freshly prepared from MC tumours, into the flank and subsequent excision of the resulting tumour after it had reached a diameter of 6–8 mm. Ten days later immune mice and control mice were challenged with varying numbers of cells by s.c. injection of test cells into the opposite flank. The relative growth rates of the subsequent tumours in the 2 groups of mice were determined by measuring with calipers the maximum tumour diameter, one at right-angles to that, and calculating the mean tumour diameter. Highly antigenic tumours showed a considerably reduced growth rate in the immunized group, although at challenge doses used in these experiments tumours grew in all mice. Immunogenicity was expressed as the *antigenic ratio* (Basombrio, 1970):

$$\frac{\text{mean tumour diameter in unimmunized mice}}{\text{mean tumour diameter in immune mice}}$$

at 16 days after tumour challenge. An antigenic ratio $>1{\cdot}0$ indicates protection.

*Antisera and serological techniques.*—The amount of H-2 on cell suspensions was assayed by absorbing anti-H-2 alloantisera with doubling dilutions of cells and testing the residual cytotoxicity by $^{51}Cr$ release from appropriately labelled lymph-node cells. Antisera (Searle, High Wycombe, England) were prepared by immunization of mice congenic for H-2: (anti H-$2^b$: B10.BR anti-B10; anti-H-$2^k$: B10 anti-B10.BR).

The relative amount of surface antigen was calculated by determining the number of cells required to absorb 50% of cytotoxic activity from the antiserum dilution used. The ratio of this number to the number of cells used as standard required to absorb 50% of cytotoxic activity was calculated. The reciprocal of this number was taken as the relative amount of surface antigen. The tumour used as standard (relative amount of antigen=1·0) was B10/MC 9. Details of this procedure are as previously described (Kohler *et al.*, 1977).

### RESULTS

### *Contamination of tumour cell suspensions by host cells*

We measured the amount of H-2 contributed by host-cell infiltration of the tumour mass to cell suspensions prepared directly from the tumour mass, and after varying numbers of passages *in vitro*. To do this, B10 or B10.BR tumours were grown in (B10×) F1 mice and cell suspensions prepared from them were assayed for ability to absorb anti-H-$2^k$ or anti-H-$2^b$ sera. This was done with cell suspensions of tumours from the animal and after various transfers in tissue culture. Absorption of B10.BR anti-B10 (anti-H-$2^b$) serum with B10/MC 6A/4 tumour-cell suspensions after varying numbers of passages *in vitro* is shown in Fig. 1(a). Cell suspensions of tumours taken directly from the animal and after 24 h *in vitro* absorb a similar amount of anti-H-$2^b$ activity. After 3 passages *in vitro* the same number of cells absorb about half as much. A similar absorption capacity was seen after 6 passages *in vitro*. Fig. 1(b) shows absorption of B10 anti-B10.BR (anti-H-$2^k$) serum with the same cell suspensions. Those made directly after removing the tumour from the animal or after 24 h in tissue culture absorb anti-H-$2^k$ activity, indicating contamination by host cells. After 3 passages *in vitro* this absorbing capacity disappeared. Results of similar experiments with B10.BR/MC 9/3 tumour suspensions are shown in Fig. 1(c) (anti-H-$2^b$ serum) and in Fig. 1(d) (anti-H-$2^k$ serum). Results similar to those for B10/MC 6A/4 suspensions were found; cell suspensions made soon after tumour excision after one or 2 passages *in vitro* absorbed both anti-H-$2^b$ and anti-H-$2^k$

FIG. 1. Absorption of anti-H-$2^b$ or anti-H-$2^k$ sera by B10/MC 6A/4 or B10.BR/MC 9/3 after growing in B10 × B10.BR hybrid mice.

● — — ● Cell suspension direct from tumour mass.

○ - - - ○ 25 h (1 passage)

□ - - - □ 3 passages } period *in vitro*.

△ - - - △ 6 passages

● Level of complement background subtracted from antisera cytotoxicity.

TABLE.—*Rank Order of Immunogenicities of B10 MC Sarcomas at Various Challenge Doses. Antigenic Ratios in Parentheses*

| | Challenge dose | | |
|---|---|---|---|
| | $2 \times 10^5$ | $1 \times 10^6$ | $2 \cdot 5 \times 10^6$ |
| | MC 6A (10·0) | MC 6B (9·9) | MC 6B (7·25) |
| | MC 6B ( 5·0) | MC 6A (7·6) | MC 6A (3·9) |
| | MC 9 (3·2) | MC 2A (5·3) | MC 12 (3·3) |
| | MC 5 (1·0) | MC 14 (4·4) | MC 13 (2·5) |
| | | MC 12 (4·3) | MC 2A (2·3) |
| | | MC 13 (4·0) | MC 14 (2·2) |
| | | MC 4 (3·4) | MC 7A (2·0) |
| | | MC 9 (2·5) | MC 8 (1·7) |
| | | MC 7A (2·4) | MC 9 (1·7) |
| Strongly antigenic | | MC 8 (2·2) | MC 4 (1·5) |
| Weakly antigenic | | MC 5 (1·3) | MC 5 (1·0) |

activity, whereas after 3 passages *in vitro* absorption of antibody directed against the haplotype not found on the tumour cells themselves disappears. After removal of host cells, the amount of H-2 expressed on several MC sarcoma lines remained constant for at least 23 passages *in vitro.*

*Immunogenicity of MC-induced B10 sarcomas*

The antigenicity of each tumour was defined in terms of its antigenic ratio, the mean tumour diameter in the controls divided by that in immunized animals at 16 days after challenge. In the Table the antigenic ratios of the collection of tumours from several sets of experiments are shown. While the values for a given tumour may vary, the rank order of the tumours remains roughly the same.

*Measurement of H-2 on MC-induced sarcomas*

In order to quantitate and compare the amount of H-$2^b$ antigens on the 11 different MC-induced sarcomas, we needed to know the reproducibility of our quantitative absorption assay. To find this out we measured the amount of H-$2^b$ on 2 tumours (B10/MC 6B/4/4 and B10/MC 5/4/4) in 3 separate absorption experiments. The antiserum and complement dilutions, $^{51}$Cr-labelled target cells and medium were made independently for each experiment. Only the tumour suspensions were prepared in one batch, each portion of cells used for the first tube in the absorption series being counted separately.

It was evident that the relative amount of H-2 on each tumour, compared with either B10 LNC or with each other, was fairly constant from one experiment to another. On the other hand, the number of cells required to absorb 50% of the cytotoxic activity from the diluted antiserum varies as much as 2-fold between experiments. This result indicated that the quantity of H-2 could only be reliably compared and normalized to LNC among suspensions in the same experiment, *i.e.* with the same serum and complement dilutions.

In a single experiment, we measured the amount of H-2 on a set of 11 different MC-induced sarcomas from B10 mice passed 3 times *in vitro*. Immunogenicity assays had been previously performed and antigenic ratios calculated for each tumour. The relative amount of H-$2^b$ *vs* the antigenic ratio was plotted, each tumour being represented by a point (Fig. 2).

Two facts are apparent. The first is that the tumours vary greatly in their degree of immunogenicity. The second is that they vary greatly in the expression of H-$2^b$. For most of the tumours there is no obvious correlation between the amount of H-$2^b$ and their degree of immunogenicity. However, the 2 most strongly immunogenic tumours do express relatively little measurable H-2, while the most weakly immunogenic tumour expresses a fair

FIG. 2.—Relationship between immunogenicity expressed as antigenic ratio and the relative amount of H-2 on 11 MC sarcomas. H-2 quantity related to the amount present on B10/MC 9. Challenge dose of $10^6$ cells in rejection assay.

amount. All other weakly immunogenic and the moderately immunogenic tumours express a great range of amounts of $H\text{-}2^b$.

### DISCUSSION

The antigens that appear on tumour cells are baffling by their diversity. The idea that these antigens are related to another diverse set, the major histocompatibility antigens, is appealing, and various kinds of evidence have been produced in support of it. One of the kinds of antigen discussed in this relationship is the set of tumour-specific transplantation antigens of methylcholanthrene-induced sarcomas of mice. Haywood and McKhann (1971) investigated this relationship with 5 tumours of C3H mice, by assaying them for the amount of H-2 and for their immunogenicity. In fact they showed that 3 of them were non-immunogenic and had much H-2, while the 2 immunogenic ones had less H-2. From this they suggested that there is an inverse relationship between these 2 quantities. The implication was that the two sets of antigens are carried by molecules that are closely enough related to compete for expression on the cell membrane.

Our attempts to test this relationship with a set of 11 methylcholanthrene-induced sarcomas of B10 mice have shown no clear correlation of the amount of H-2 and tumour-specific immunogenicity. To quantitate H-2 on the tumour cells we found it essential to eliminate host cells that infiltrate the tumour mass and may contribute much of the H-2 measured on the cell suspension prepared from it. To do this, we had to passage the cells 3 times *in vitro*. We did confirm for several tumours that cells so passed retained specific antigenicity.

How reliable are the data in Fig. 2? It is important to consider this question carefully, for we do not have to remove many points from the graph to leave a fairly good inverse relationship between immunogenicity (measured by antigenic ratio) and amount of H-2. In fact, if the data from Tumours 7(A), 8, and 12 are ignored (only 3/11 tumours), we could make a good case for this relationship. We can see, however, no justification for doing this. While the absolute amounts of H-2 measured on the various tumours do vary from one experiment to another, their ranking on the basis of amount of H-2 stays fairly constant. The same holds true for the measurements of immunogenicity.

We think that the question whether tumour-associated antigens or tumour-specific transplantation antigens are like major histocompatibility antigens will not be answered by experiments of this kind. An answer will come from these experiments that examine this relationship more directly, either by serological or chemical techniques. In the end only comparison of the isolated molecules can settle this question.

### REFERENCES

BASOMBRIO, M. A. (1970) Search for Common Antigenicities among 25 Sarcomas Induced by Methylcholanthrene. *J. natn. Cancer Inst.*, **41**, 1411.

CIKES, M., FRIBERG, S., JR. & KLEIN, G. (1973) Progressive Loss of H-2 Antigens with Concomitant Increase of Cell-surface Antigen(s) Determined by Moloney Leukaemia Virus in Cultured Murine Lymphomas. *J. natn. Cancer Inst.*, **50**, 347.

EVANS, R. (1972) Macrophages in Syngeneic Animal Tumours. *Transplantation*, **14**, 468.

GARRIDO, F., FESTENSTEIN, H. & SCHIRRMACHER, V. (1976) Further Evidence for Derepression of H-2 and Ia-like Specificities of Foreign Haplotypes in Mouse Tumour Cell Lines. *Nature*, **261**, 705.

HAYWOOD, G. R. & MCKHANN, C. F. (1971) Antigenic Specificities on Murine Sarcoma Cells. Reciprocal Relationship between Normal Transplantation Antigens (H-2) and Tumour-specific Immunogenicity. *J. exp. Med.*, **133**, 1171.

KERBEL, R. S., PROSS, H. F. & ELLIOT, E. V. (1975) Origin and Partial Characterization of Fc-receptor Bearing Cells Found within Experimental Carcinomas and Sarcomas. *Int. J. Cancer*, **15**, 918.

KOHLER, G., PEARSON, T. & MILSTEIN, C. (1977) Fusion of T and B Cells. *Somatic Cell Gentet.*, **3**, 303.

LENNOX, E. S. & SIKORA, K. (1977) Tumour-specific Transplantation Antigens of Chemically Induced Tumours. In *Differentiation and Carcinogenesis*; *Advances in Pathobiology* No. 7, Eds. C. Borek and D. W. King, New York: Stratten Intercont. Book Corp. p. 68.

MARTIN, W. J., ESBER, E., COTTON, W. G. & RICE, J. M. (1973) Derepression of Alloantigens in Malignancy. Evidence for Tumour Susceptibility Alloantigens and for Possible Self Reactivity of Lymphoid Cells Active in the Microcytotoxicity Assay. *Br. J. Cancer*, **28**, Suppl. 1, 48.

MARTIN, W. J., GIPSON, T. G. & RICE, J. M. (1977) H-$2^a$ Associated Alloantigen Expressed by Several Transplacentally-induced Lung Tumours of C3Hf Mice. *Nature*, **265**, 738.

PROSS, H. F. & KERBEL, R. S. (1976) An Assessment of Intratumour Phagocytic and Surface Marker-bearing Cells in a Series of Autochthonous and Early Passaged Chemically Induced Murine Sarcomas. *J. natn. Cancer Inst.*, **57**, 1157.

TING, C. & HERBERMAN, R. B. (1971) Inverse Relationship of Polyoma Tumour-specific Cell Surface Antigen to H-2 Histocompatibility Antigens. *Nature, New Biol.*, **232**, 118.

TSAKRAKLIDES, E., SMITH, C., KERSEY, J. H. & GOOD, R. A. (1974) Transplantation Antigens (H-2) on Virally and Chemically Transformed BALB/3T3 Fibroblasts in Culture. *J. natn. Cancer Inst.*, **52**, 1499.